# Lineage-specific evolution of Methylthioalkylmalate synthases (MAMs) involved in glucosinolates biosynthesis

**DOI:** 10.3389/fpls.2015.00018

**Published:** 2015-02-03

**Authors:** Jifang Zhang, Xiaobo Wang, Feng Cheng, Jian Wu, Jianli Liang, Wencai Yang, Xiaowu Wang

**Affiliations:** ^1^Institute of Vegetables and Flowers, Chinese Academy of Agricultural SciencesBeijing, China; ^2^Beijing Key Laboratory of Growth and Developmental Regulation for Protected Vegetable Crops, Department of Vegetable Science, China Agricultural UniversityBeijing, China

**Keywords:** glucosinolates, *MAM* genes, syntenic, evolution, Brassicaceae

## Abstract

Methylthioalkylmalate synthases (MAMs) encoded by *MAM* genes are central to the diversification of the glucosinolates, which are important secondary metabolites in Brassicaceae species. However, the evolutionary pathway of *MAM* genes is poorly understood. We analyzed the phylogenetic and synteny relationships of *MAM* genes from 13 sequenced Brassicaceae species. Based on these analyses, we propose that the syntenic loci of *MAM* genes, which underwent frequent tandem duplications, divided into two independent lineage-specific evolution routes and were driven by positive selection after the divergence from *Aethionema arabicum*. In the lineage I species *Capsella rubella*, *Camelina sativa*, *Arabidopsis lyrata*, and *A. thaliana*, the *MAM* loci evolved three tandem genes encoding enzymes responsible for the biosynthesis of aliphatic glucosinolates with different carbon chain-lengths. In lineage II species, the *MAM* loci encode enzymes responsible for the biosynthesis of short-chain aliphatic glucosinolates. Our proposed model of the evolutionary pathway of *MAM* genes will be useful for understanding the specific function of these genes in Brassicaceae species.

## Introduction

Plants synthesize an immense number of defensive compounds to attack other organisms. An important model system for studying the role of chemical defenses in plants is the glucosinolates, a group of sulfur-rich secondary metabolites largely found in the plant family Brassicaceae (or Cruciferae). Glucosinolates and their degradation products play an important roles against microbial pathogens and herbivorous insects (Kroymann et al., 2003; Clay et al., 2009), and are responsible for the special flavors of *Brassica* vegetables such as turnip (*Brassica rapa* ssp. *rapa*), broccoli (*Brassica oleracea* var. italica), and caulifiower (*B. oleracea* var. botrytis) (Schonhof et al., 2004; Padilla et al., 2007). Furthermore, glucosinolates are of particular interests because of their cancer-preventing properties to human beings. They can inhibit carcinogen activation (Hecht, 2000; Nakajima et al., 2001) and carcinogenesis by triggering cell cycle arrest and stimulating apoptosis (Wittstock et al., 2003; Hayes et al., 2008).

Glucosinolates are derived from amino acids, and can be classified as aliphatic, aromatic, or indole glucosinolates according to their precursor amino acids (Halkier and Gershenzon, 2006; Sonderby et al., 2010). There are three independent processes for glucosinolate biosynthesis: (1) chain elongation of the precursor amino acid; (2) formation of the core structure; and (3) side chain modification. Differences in the degree of elongation and modification of the side chains lead to diverse glucosinolate structures. To date, more than 30 different glucosinolates have been identified in *A. thaliana* (Kliebenstein et al., 2001; Windsor et al., 2005).

Methylthioalkylmalate synthases (MAMs) are involved in amino acid chain elongation, and give rise to glucosinolates with diverse chain-lengths during the biosynthesis of methionine-derived glucosinolates in *A*. *thaliana* (Kliebenstein et al., 2001). It is thought that *MAM* genes are derived from isopropylmalate synthase genes (*IPMS)*, which encode the enzymes that catalyze the first step of leucine biosynthesis (De Kraker et al., 2007). *MAM* genes are often found as clusters of tandem arrays but differentiated genes in the genome. In *A. thaliana*, the configuration of the *MAM* cluster comprises three genes in one tandem array, *AtMAM2*, *AtMAM1*, and *AtMAM3* (*AtMAM-L*), though there are variations among accessions because of gene deletion or conversion events (Kroymann et al., 2003). AtMAM3 catalyzes the formation of all aliphatic glucosinolates, especially long-chain glucosinolates (6C, 7C, and 8C) in *Arabidopsis* (Textor et al., 2007). AtMAM2 and AtMAM1 catalyze the formation of short-chain aliphatic glucosinolates (3C and 4C) (Kliebenstein et al., 2001; Kroymann et al., 2003; Textor et al., 2004). In *A. lyrata*, which produces mainly 3C Met-derived glucosinolates and lower levels of long-chain glucosinolates (Windsor et al., 2005), the *MAM* cluster contains three directly repeated paralogous sequences (*MAMa*, *MAMb*, and *MAMc*). MAMa controls the first Met chain extension, MAMb is responsible for the long-chain Met-glucosinolates, and the function of MAMc is unclear (Benderoth et al., 2006).

The Brassicaceae is a medium-sized family that contains 338 genera and 3709 species, and includes many economically important crops (Warwick et al., 2006). This family can be split into two major groups: the *Aethionema* group, and the core group (Franzke et al., 2011). Three major lineages (lineages I, II, and III) have been proposed in the core group, based on the sequences of the chloroplast gene *ndhF* and supported by subsequent studies (Beilstein et al., 2006; Koch et al., 2007; Couvreur et al., 2010). The core group has undergone three ancient whole-genome duplication (WGD) events (Franzke et al., 2011). These events have played a crucial role in the genetic diversification and species radiation of lineages in Brassicaceae. Furthermore, whole-genome triplication events occurred in *Brassica* (Br-α), *Leavenworthia alabamica* (La-α), and *Camelina sativa* (Cs-α), as determined by analyses of their recently sequenced genomes (Haudry et al., 2013; Slotte et al., 2013; Cheng et al., 2014). These genome duplication events followed by gene losses during diploidization resulted in very complex relationships among the duplicated *MAM* genes in Brassicaceae (Benderoth et al., 2006; Sonderby et al., 2010). Furthermore, the local tandem duplication (TD) events occurred frequently at the *MAM* loci after genome duplication, making the relationship even more complicated. Although it is very challenging to clarify the evolutionary history of *MAM* genes in Brassicaceae, it is important to explore their evolution to deduce the functions of the diversified and duplicated *MAM* genes in extant Brassicaceae species.

Fortunately, the genomes of 13 crucifer species have been completely or partially sequenced, providing the opportunity to clarify the evolution of *MAM* genes. The sequenced crucifer species include: (1) five species from lineage I, they are the model plant *A. thaliana* (Initiative, 2000), *A. lyrata* (Hu et al., 2011), *Capsella rubella* (Slotte et al., 2013), *L. alabamica* (Haudry et al., 2013), and *Camelina sativa* (Kagale et al., 2014); (2) seven species from lineage II, *B. rapa* (Wang et al., 2011b), *Thellungiella salsuginea* (Wu et al., 2012), *Schrenkiella parvula* (synonym of *Thellungiella parvula*) (Dassanayake et al., 2011), *Thellungiella halophila* (Yang et al., 2013b), *Sisymbrium irio* (Haudry et al., 2013), *B. oleracea* (Liu et al., 2014), and *Raphanus sativus* (Kitashiba et al., 2014); (3) *Aethionema arabicum*, an early branching sister group to the core Brassicaceae group (Haudry et al., 2013). And there is no sequenced lineage III species available to date. Here, we took advantage of the whole genome sequences to investigate the evolution and diversification of *MAM* genes in Brassicaceae. Our analyses revealed the lineage-specific evolutionary routes that have led to the diversified structure of aliphatic glucosinolates.

## Materials and methods

### Sources of genome data

*B. rapa* gene sequences for synteny analyses were obtained from BRAD (V1.5; http://brassicadb.org) (Cheng et al., 2012). Gene and genome data sets for *A. thaliana* were downloaded from The Arabidopsis Information Resource (TAIR9; http://www.Arabidopsis.org/index.jsp). The genomic dataset for *A. lyrata* was downloaded from the Joint Genome Initiative database (Gene model 6; http://genome.jgi-psf.org/Araly1/Araly1.home.html) (Hu et al., 2011). *S. parvula* and *T. salsuginea* datasets were obtained from Dassanayake et al. (2011) and Wu et al. (2012). Three *MAM* genes have been annotated in the genomes *S. parvula* and *T. salsuginea*, with two of them located at a tandem array. Gene and genome data for *T. halophila* were obtained from Yang et al. (2013b). The *L. alabamica*, *S. irio* and *A. arabicum* genomic datasets were obtained from Haudry et al. (2013). The *C. sativa* genomic dataset was obtained from Kagale et al. (2014) with nine syntenic and three non-syntenic *MAM* genes annotated. *B. oleracea* genomic was obtained from Liu et al. (2014), which has more than six annotated *MAM* genes. The *R. sativus* genomic dataset was obtained from Kitashiba et al. (2014) and contains at least two *MAM* genes.

### Syntenic ortholog determination

Multi-syntenic orthologs between *A. thaliana* and other sequenced Brassicaceae species such as *B. rapa*, *A. lyrata*, *S. parvula*, *T. salsuginea*, *T. halophila*, *L. alabamica*, *S. irio*, *A. arabicum*, *C. sativa*, *B. oleracea*, and *R. sativus* were identified with the tool SynOrths and through the following URL: http://brassicadb.org/brad/searchSyntenytPCK.php.

### Phylogenetic analyses and sequence features

The full-length sequences of the MAM proteins encoded by genes in the 12 sequenced Brassicaceae species were aligned using Clustal W with default parameters (Larkin et al., 2007). A phylogenetic tree was constructed using the neighbor-joining method with Mega version 5.0 software (Tamura et al., 2011). Support for the topology was estimated from 1000 bootstrap replicates, and nodes occurring in less than 50% of the replicates were collapsed. Gene structures were determined by comparing coding and genomic sequences among *MAM* genes, based on information obtained from the Gene Structure Display Server (GSDS, Guo et al., 2007).

### Motif identification

MEME version 4.9.1 (Bailey et al., 2009) was used to identify the conserved motifs of syntenic MAM proteins in the sequenced Brassicaceae species. The parameters for the analysis were as follows: number of repetitions, 0 or 1; maximum number of motifs, 9; and optimum motif width, 6–200. The MAST program (Bailey and Gribskov, 1998) was used to search for each of the motifs in MAM sequences. The MEME program was used to extract each motif sequence from the syntenic *MAM* genes. The motifs were further characterized using the Conserved Domain Search Service (Marchler-Bauer et al., 2011).

### Tests for selective pressure

Pairwise alignments of each motif in syntenic *MAM* genes were made using ClustalX2 (Larkin et al., 2007), with the corresponding protein sequences as the alignment guides. Gaps in the alignments were removed. The analysis of synonymous (Ks) and non-synonymous (Ka) substitution rates was carried out using the KaKs Calculator version 1.2 (Zhang et al., 2006). This program implements several candidate models of codon substitution in a maximum likelihood framework. We used the MS method to estimate Ka and Ks values with default parameters.

## Results

### Identification of *MAM* genes in genomes of Brassicaceae

Shared synteny describes genomic fragments in different species that are inherited from a common ancestor (Lyons et al., 2008). Syntenic genes are orthologs that located at these syntenic fragments, and they often share similar functions. We identified the *MAM* genes in the 13 sequenced Brassicaceae species according to their gene annotation information and their gene synteny relationship to the model plant species *A. thaliana* (http://brassicadb.org/brad/searchSyntenytPCK.php). *B. rapa* contains seven *MAM* genes including five syntenic and two non-syntenic ones (Wang et al., 2011a). We found one syntenic gene in *L. alabamica* (*LaMAM*), two syntenic genes in *T. halophila*, and four syntenic genes in each of *A. arabicum* and *C. rubella* (Table 1). No *MAM* genes were identified in *S. irio*. Table 1 lists the information of *MAM* genes in each genome of the 13 Brassicaceae species.

**Table 1:** ***MAM* genes identified in 13 species from Brassicaceae**.

**Species**	**Gene ID**	**References**
*A. arabicum*	*AaMAM-1* (AA_scaffold229_161), *AaMAM-2* (AA_scaffold229_162), *AaMAM-3* (AA_scaffold229_165), *AaMAM-4* (AA_scaffold229_166)	Haudry et al., 2013
*C*. *rubella*	*CrMAM-1* (Carubv10003425m), *CrMAM-2* (Carubv10003885m), *CrMAM-3* (Carubv10003624m), *CrMAM-4* (Carubv10003002m)	Slotte et al., 2013
*L. alabamica*	*LaMAM* (LA_scaffold763_4)	Haudry et al., 2013
*C. sativa*	*CsMAM-1* (Csa08g017360.1), *CsMAM-2* (Csa08g017370.1), *CsMAM-3* (Csa08g017380.1), *CsMAM-4* (Csa13g027610.1), *CsMAM-5* (Csa13g027620.1), *CsMAM-6* (Csa13g027630.1), *CsMAM-7* (Csa20g037920.1), *CsMAM-8* (Csa20g037930.1), *CsMAM-9* (Csa20g037940.1), *CsMAM-10* (Csa07g050930.1)^*^, *CsMAM-11* (Csa09g085060.1)^*^, *CsMAM-12* (Csa16g042520.1)^*^	Kagale et al., 2014
*A. thaliana*	*AtMAM1*, *AtMAM2*, *AtMAM3*	Kroymann et al., 2003
*A. lyrata*	*MAMa*, *MAMb*, *MAMc*	Benderoth et al., 2006
*T. halophila*	*ThMAM-1* (Thhalv10003976m), *ThMAM-2* (Thhalv10004072m)	Yang et al., 2013a
*T. salsuginea*	*TsMAM-1* (Tsa2g29250), *TsMAM-2* (Tsa2g29260), *TsMAM-3* (Tsa2g23840)^*^	Wu et al., 2012
*S. parvula*	*SpMAM-1* (C0003_00624), *SpMAM-2* (C0003_00625), *SpMAM-3* (C0011_00271)^*^	Dassanayake et al., 2011
*S. irio*	—	Haudry et al., 2013
*B. rapa*	*BrMAM-1* (Bra029355), *BrMAM-2* (Bra029356), *BrMAM-3* (Bra013007), *BrMAM-4* (Bra013009), *BrMAM-5* (Bra013011), *BrMAM-6* (Bra018524)^*^ *BrMAM-7* (Bra021947)^*^	Wang et al., 2011a
*B. oleracea*	*BoMAM-1* (Bol017070), *BoMAM-2* (Bol017071), *BoMAM-3* (Bol020647), *BoMAM-4* (Bol020646), *BoMAM-5* (Bol020644), *BoMAM-6* (Bol037823)^*^, *BoMAM-7* (Bol040636)^*^	Liu et al., 2014
*R. sativus*	*RsMAM-1* (Rsa10019680), *RsMAM-2* (Rsa10018392)	Kitashiba et al., 2014

Asterisk (

* *) indicates non-syntenic MAM genes*.

**Figure 1:**
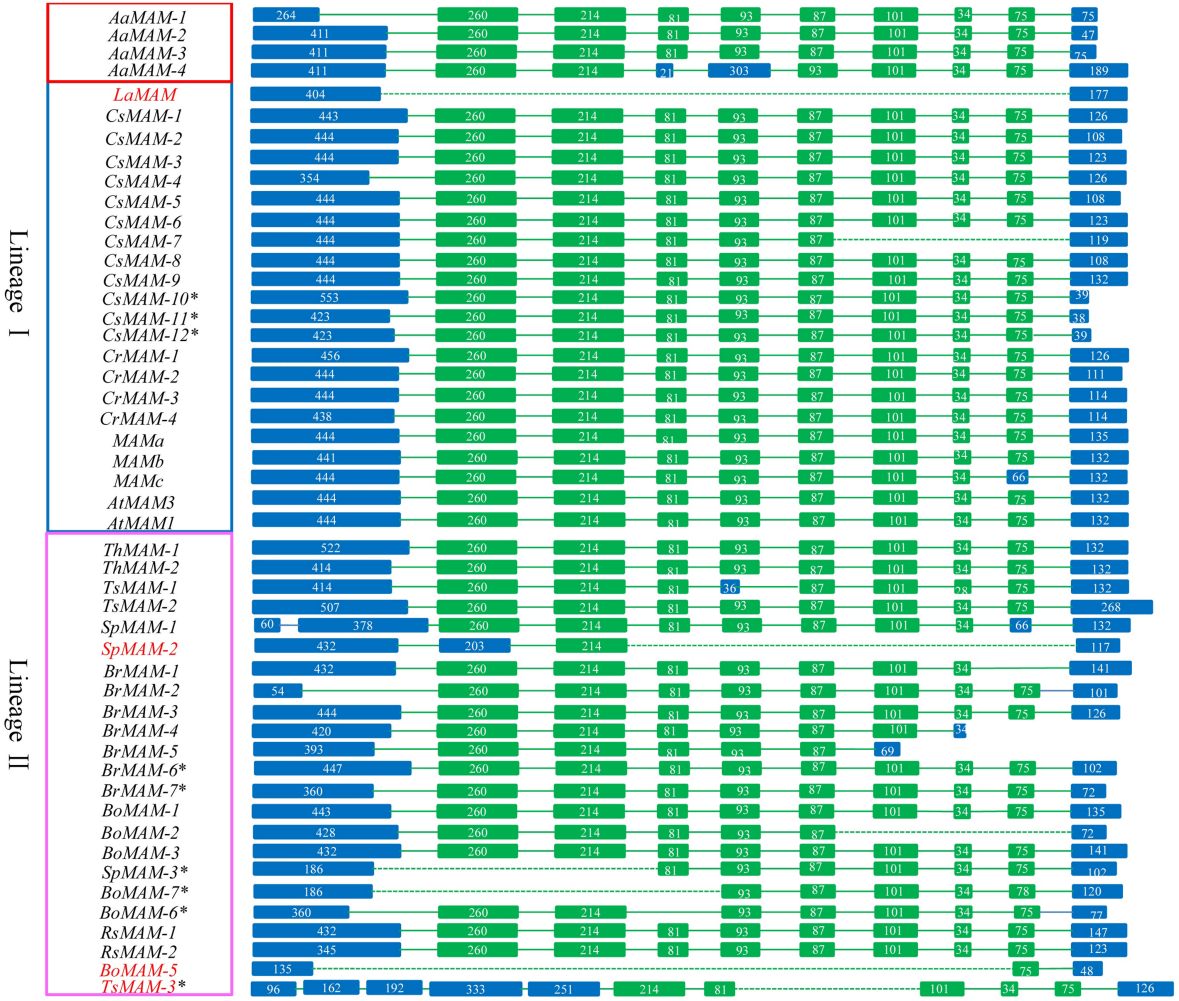
**Schematic diagram of structure of *MAM* genes in 12 sequenced Brassicaceae species (excluding *S. irio*)**. Green blocks, conserved exons; blue blocks, variable exons. Most *MAMs* share the same gene structure, except for *LaMAM* in lineage I and *SpMAM-2*, *BoMAM-5*, and *TsMAM-3* in lineage II (gene ID shown in red). Dashed lines indicate absence of exons from the region, compared with corresponding region in *A. thaliana*. Numbers in blocks indicate exon length. Genes shown in red, blue, and pink blocks (on left) are from *A. arabicum*, lineage I species, and lineage II species, respectively.

We analyzed the structures of the *MAM* genes in these Brassicaceae species. Most *MAM* genes shared conserved gene structures. Four *MAM* genes were very short, lost some conserved domains or were heavily differentiated to the other *MAM* genes. Compared with the majority of *MAM* genes, *LaMAM* in lineage I and *SpMAM-2* and *BoMAM-5* in lineage II had fewer exons, and *TsMAM-3* had a different gene structure (Figure 1).

### Two major groups of *MAM* genes evolved independently in core Brassicaceae

**Figure 2:**
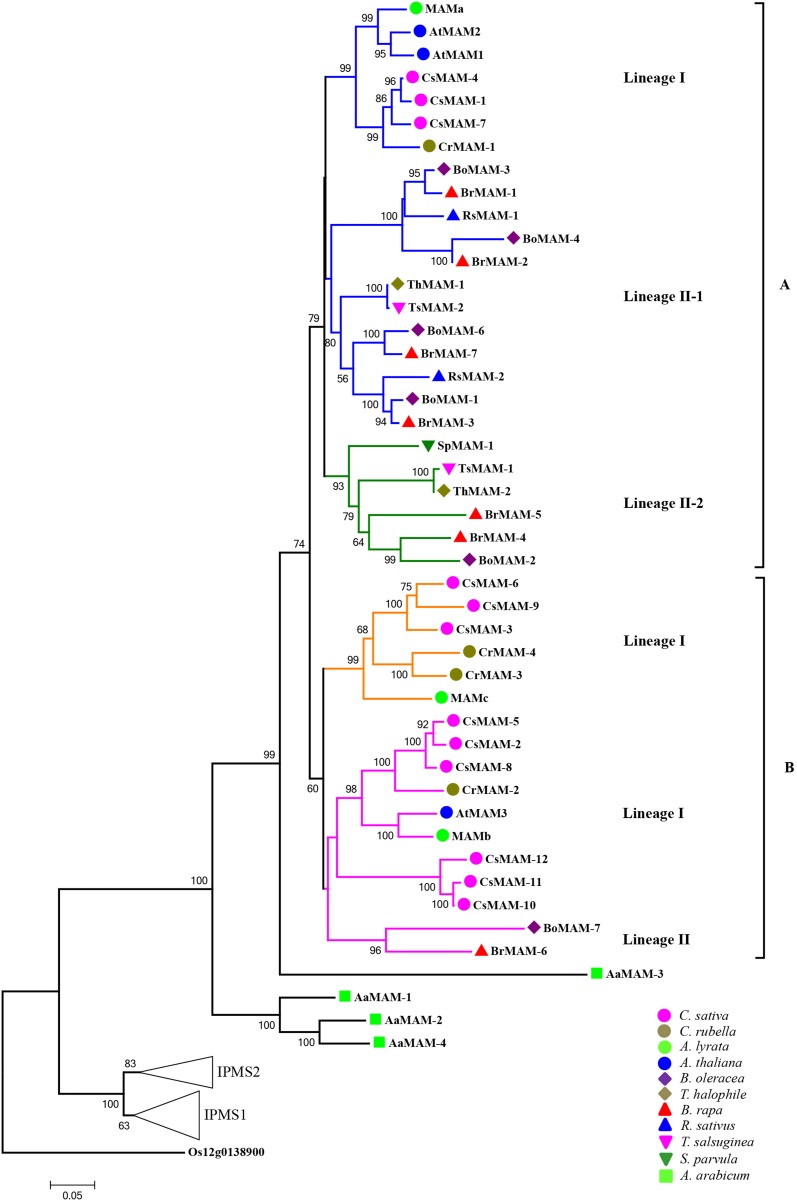
**Phylogeny relationships of *MAM* genes in Brassicaceae, based on protein sequences of *MAM* genes**. Phylogenetic tree was constructed using the full-length sequences of MAM and IPMS proteins encoded by genes in 13 sequenced Brassicaceae species (excluding *LaMAM*, *SpMAM-2*, *BoMAM-5*, and *TsMAM-3*). The rice gene Os12g0138900 encoded isopropylmalate synthase was used as the outgroup to build the phylogenetic tree. Numbers on branches indicate percentage bootstrap support (1000 replicates). Genes in the core Brassicaceae group formed two major groups (A and B) representing four clades (shown as colored branches). Triangles labeled *IPMS1* and *IPMS2* represent evolution of *IPMS* in Brassicaceae (see Supplemental data for more detail).

Using the neighbor-joining method, we constructed a phylogenetic tree for *MAM* genes, based on the sequences of MAM proteins in the 12 sequenced species (exclude *S. irio*). This analysis excluded four short/different genes, *LaMAM*, *SpMAM-2*, *BoMAM-5*, and *TsMAM-3*, but included *IPMS* genes (predicted by synteny analyses with *AtIPMS1* and *AtIPMS2* of *Arabidopsis*). Rice *IPMS* served as the outgroup (Figure 2).

The phylogenetic tree showed that all of the *A. arabicum MAM* genes were clustered with the same *A. arabicum MAM* gene, *AaMAM-3*. The rest of the *MAM* genes in the sequenced species in the core Brassicaceae group originated from *AaMAM-3*, and formed two major relatively distinct groups, group A and group B. Each group contained one or more *MAM* genes from the same species, suggesting that duplication and gene diversification had occurred frequently in the subsequent evolution of *MAM* genes.

Group A included one clade of *MAM* genes from lineage I species (Figure 2, blue branches) and two clades of *MAM* genes from lineage II species (Figure 2, blue and green branches). Three previously identified *Arabidopsis* genes, *AtMAM1*, *AtMAM2*, and *MAMa*, which are responsible for short-chain Met-derived glucosinolate biosynthesis, were all in group A. Three *C. sativa* genes and one *C. rubella* gene clustered with the Arabidopsis *MAM* genes. The other two clades of *MAM* homologs from lineage II species (lineage II-1 and lineage II-2) clustered together with the lineage I clade, suggesting that they share the same or a similar function as that of *Arabidopsis MAM* genes, which encode enzymes that biosynthesize short-chain aliphatic glucosinolates. Furthermore, in each lineage II group, the *MAM* genes in the *Brassica* genus were more similar to those in the *Thellungiella* genus than to those in the *Arabidopsis* genus. This result is consistent with the finding that *Brassiceae* and *Arabidopsis* ancestors diverged before the split of *Brassiceae-Thellungiella* (Cheng et al., 2013).

Group B comprised *MAM* genes from the same lineage I species (Figure 2, yellow and pink branches) and two non-syntenic *MAM* genes from *B. rapa* and *B. oleracea* (Figure 2, pink branches). AtMAM3 and MAMb (pink clade) encode enzymes that biosynthesize long-chain glucosinolates, suggesting that the other *MAM* genes in this clade encode enzymes with similar functions. The function of *MAMc* (Figure 2, yellow clade) remains unclear (Benderoth et al., 2006). All of the *MAM* and *IPMS* genes in Brassicaceae clustered together, suggesting that they share a common ancestral gene originating from monocots.

### *MAM* ancestral locus is a tandem gene array and shares clear synteny among Brassicaceae species

**Figure 3:**
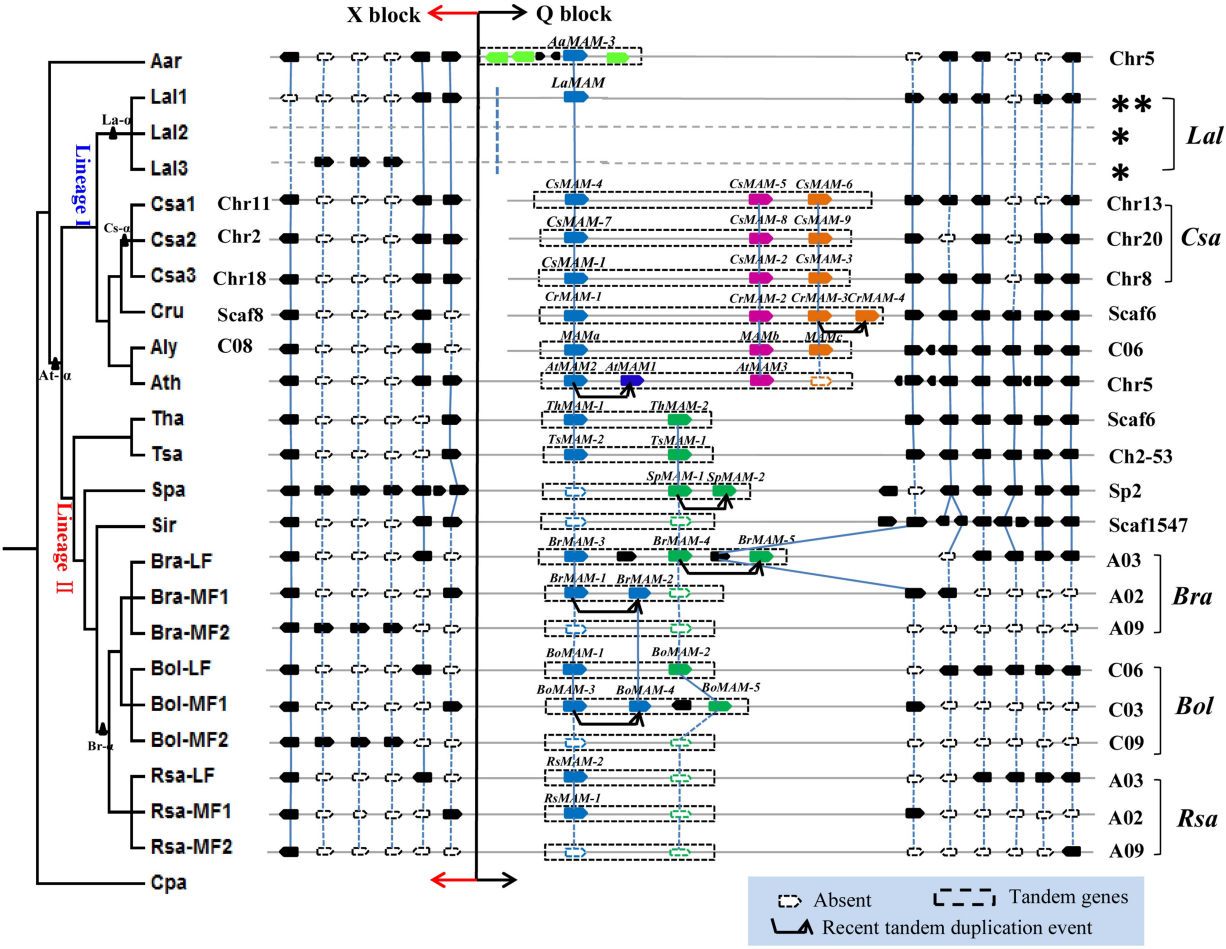
**Synteny analyses of *MAM* region in the family Brassicaceae showing lineage-specific rearrangements, local tandem duplications, and diverse patterns of gene loss**. Phylogenetic tree on the left represent the evolutionary relationships among 13 sequenced Brassicaceae species. Black triangles mark whole genome duplication (At-α) and whole genome triplication (La-α, Cs-α, and Br-α) events. *MAM* region shows conserved synteny across all sequenced Brassicaceae genomes. *MAM* tandem genes located at the end of Q Block, linked to X Block, are highlighted in colored blocks; blue and green blocks show genes responsible for elongation of short-chain glucosinolates, pink blocks show genes responsible for long-chain glucosinolate biosynthesis, orange blocks show genes whose function is unclear. Genes shown as solid and hollow (white) symbols in *MAM* region are present and absent, respectively. Chromosomal location of *MAM* region in each species is shown on the right or left. Single asterisk indicates that sub-genomic location of *LaMAM* region could not be determined because of scarce syntenic genes in this region. Dashed line at junction between X and Q Blocks and double asterisk on upper right of *L. alabamica* indicates that it is unclear whether these two blocks are on the same chromosome or not.

To assess the contributions of polyploidy and tandem gene duplications to *MAM* gene diversification, we conducted detailed synteny analyses within these 13 Brassicaceae genomes using the tool SynOrths (Cheng et al., 2012). The *MAM* region showed conserved synteny across all of the sequenced Brassicaceae genomes (Figure 3), consistent with other studies demonstrating extensive synteny among cruciferous genomes (Rossberg et al., 2001; Boivin et al., 2004; Kuittinen et al., 2004; Cheng et al., 2013).

Three tandem *MAM* genes, with the same gene order and orientation, were located in the conserved syntenic region of most lineage I species, except for *L. alabamica*, which had only one *LaMAM*. The other two *MAM* homologs would have been lost from *L. alabamica* either after its divergence from *A. arabicum*, or more recently. Based on our phylogenic analysis, these three tandem genes were assumed to be responsible for the different chain lengths of glucosinolates (Figure 3, labeled with different colors). The genomes of species in Brassicaceae comprise 24 genomic blocks (A–X, also known as ancestral karyotypes, AK) (Parkin et al., 2005; Schranz et al., 2006). The X and Q Blocks are located on different chromosomes or scaffolds in *C. sativa*, *C. rubella*, and *A. lyrata*, but on the same chromosome in *A. thaliana* (Figure 3). These patterns of arrangement suggested that major chromosome rearrangements have occurred near the *MAM* loci. However, we could not identify whether the Q and X Blocks in *L. alabamica* were present on the same chromosome or not, because several different scaffolds were involved in this region. Furthermore, apart from the local TD event in *A. thaliana* that led to *AtMAM1* and *AtMAM2* (Benderoth et al., 2006), we found another recent TD event in *C. rubella* that led to the two homologous genes *CrMAM-3* and *CrMAM-4*.

Two types of *MAM* genes with minor sequence variations (Figure 2, lineage II-1, lineage II-2) were located in the conserved syntenic region of most lineage II species. This syntenic region in lineage II species differed from the *MAM* loci of lineage I species. *SpMAM-1* from *S. parvula* clustered with *TsMAM-1* and *ThMAM-2*, but not with *TsMAM-2* and *ThMAM-1* (Figure 2), indicating that the ancestral type (blue) had been lost, and that *SpMAM-1* gained the function of the *MAM* ancestral gene. In *S. irio*, there were no *MAM* genes in this syntenic region. The phylogenetic tree indicated that *SpMAM-1*, *BrMAM-1*, *BrMAM-4* and *BoMAM-3* had undergone recent TD events, giving rise to more *MAM* homologous genes in *S. parvula*, *B. rapa*, and *B. oleracea*, respectively, to allow adaptation to environmental changes.

### Lineage-specific evolutionary patterns of *MAM* loci

**Figure 4:**
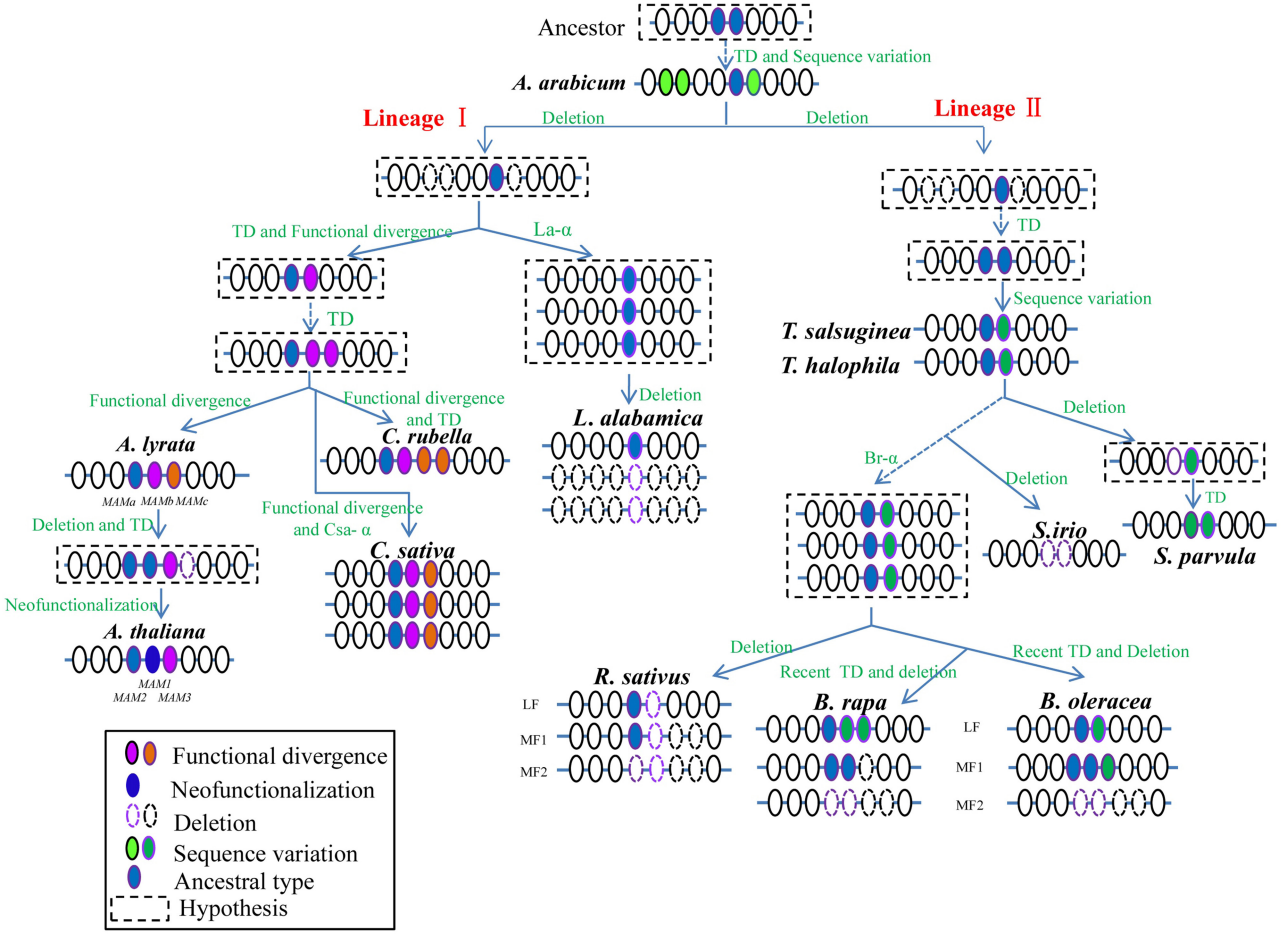
**Putative model for *MAM* loci lineage-specific evolution in Brassicaceae**. After the divergence of *A. arabicum*, the evolution of *MAM* loci proceeded via two independent lineage-specific routes: the lineage I-specific evolutionary route, and the lineage II-specific route. Colored solid circles represent *MAM* genes, white circles show their flanking genes. Changes in colors of *MAM* genes and dashed lines on flanking genes indicate specific evolutionary events. Genes within dashed lines indicate presumed process during evolution. TD, tandem duplication.

Based on the phylogeny and synteny analyses across the sequenced Brassicaceae species, we proposed a lineage-specific evolution pattern for the syntenic *MAM* loci (Figure 4). The evolution of *MAM* loci could be divided into two independent lineage-specific routes after the divergence of *A. arabicum*; a lineage I-specific route and a lineage II-specific route.

Before the separation of *A. arabicum* and the core Brassicaceae group, an ancient ancestral locus with one or two *MAM* homologs should have evolved to give rise to multiple *AaMAM* genes through several TD events. Then, *AaMAM-3* diverged from the multiple copies, forming the ancestral *MAM* gene (Figure 4, blue circles) of the core Brassicaceae species. The other three *AaMAM* homologs (Figure 4, green circles) were possibly lost from the core Brassicaceae species. The lineage-specific evolution of *MAM* loci in the core Brassicaceae group was assumed to proceed alongside the split of the three major lineages (lineages I, II, and III), possibly driven by At-α.

During the formation of the *MAM* loci in lineage I, the ancestral gene was first amplified by a TD event, followed by functional divergence to give rise to two distinct *MAM* genes (Figure 4, blue and pink). Then, the duplicated gene (Figure 4, pink circles) underwent another round of TD and functional divergence events to generate three *MAM* genes in *A. lyrata* (*MAMa*, *MAMb*, and *MAMc*) and *C. rubella*. Subsequently, in *C. rubella*, one *CrMAM* gene (Figure 4, orange circles) underwent another local TD event, leading to four *CrMAM* genes, possibly encoding proteins with three different functions. This TD may have occurred soon after the split or more recently. In *A. thaliana*, *AtMAM1*, and *AtMAM2* evolved from a *MAMa* duplication event. *AtMAM2* retained the original function, while *AtMAM1* acquired a new function (neofunctionalization) (Benderoth et al., 2006). *AtMAM3* originated from *MAMb*, and *MAMc* was lost. *C. sativa* underwent an extra whole genome triplication (WGT) event (Cs-α). In the well-preserved hexaploid genome of *C. sativa*, three clusters with a total of nine syntenic *CsMAM* genes were retained. However, in *L. alabamica*, which also underwent a WGT event (La-α), only one syntenic *LaMAM* gene was retained because of extensive gene losses during rediploidization.

Compared with the lineage I route, the lineage II route represents a different evolutionary process for *MAM* loci. After the first amplification of the ancestral gene, two copies (Figure 4, blue circles) were retained until the speciation of *T. halophila* and *T. salsuginea*, when minor sequence variations arose in the duplicated gene, but were not substantial enough to lead to functional divergence (Figure 4, green circles). When *S. parvula* diverged, the ancestral gene (Figure 4, blue circles) tended to degenerate and be lost, but the duplicated gene (Figure 4, green circles) underwent a local TD event to produce two *SpMAM* homologs. However, in *S. irio*, the two syntenic *MAM* copies were completely lost, either when it diverged from *Thellungiella* or more recently. Subsequently, when the *Brassica* genus split out, *B. rapa*, *B. oleracea*, and *R. sativus* retained various copies of *MAM* genes that were generated from WGT (Br-α) followed by biased gene lost. The fact that there are two pairs of duplicates (*BrMAM-1*/*BrMAM-2* and *BrMAM-4*/*BrMAM-5*) in the LF and MF1 subgenomes of *B. rapa*, and one pair (*BoMAM-3*/*BoMAM-4*) in the LF subgenome of *B. oleracea* further suggested that recent TD events occurred after Br-α.

### Different structures of proteins encoded by syntenic *MAM* genes

**Figure 5:**
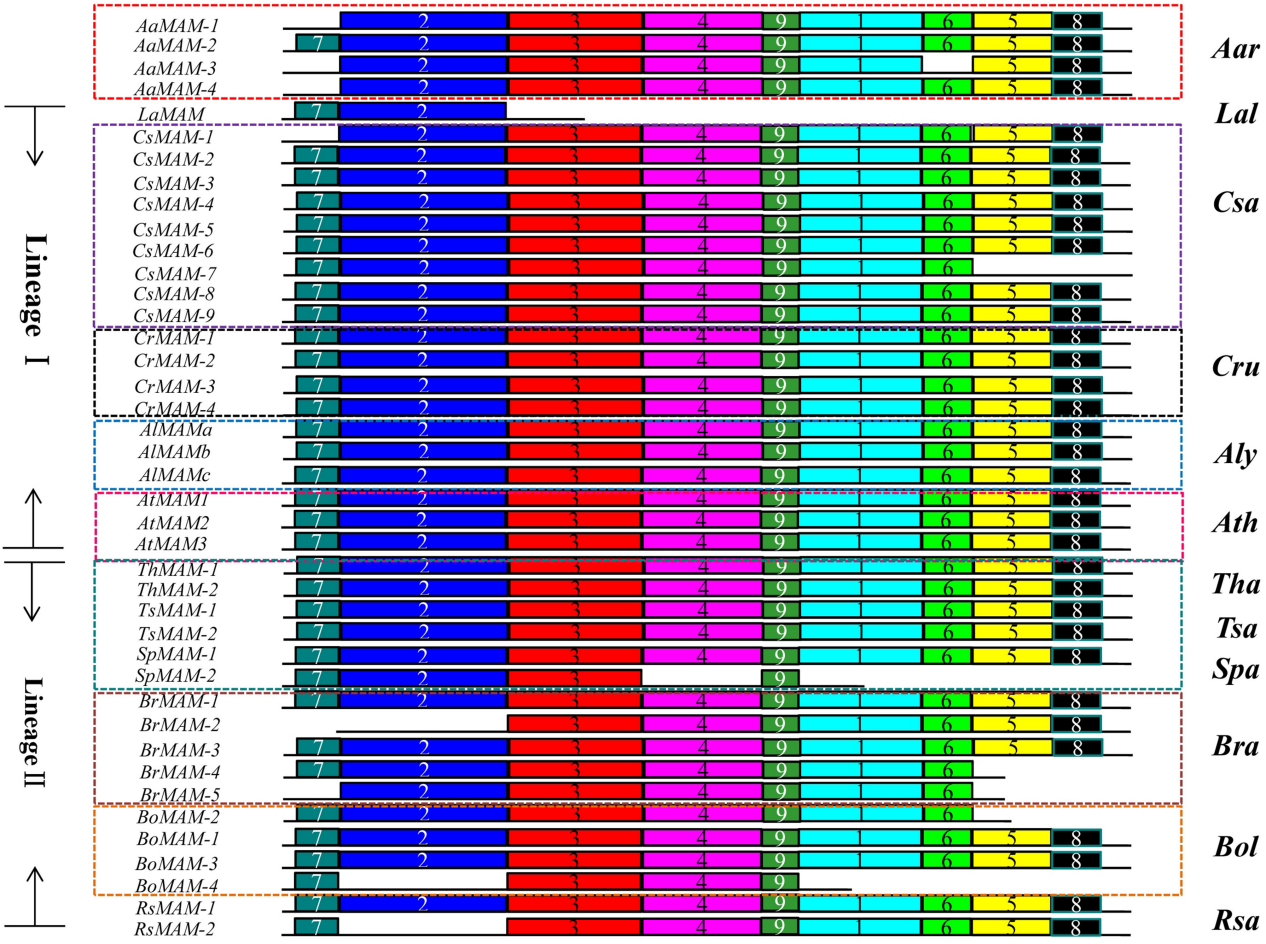
**Motif structures of syntenic *MAM* genes in Brassicaceae**. *MAM* genes in lineage I encode proteins with nine intact motifs (except for *LaMAM*, *CsMAM-1*, and *CsMAM-7*). In lineage II species, seven genes in four species encode proteins with that lack some motifs, indicating that the proteins encoded by genes at the *MAM* loci underwent rapid functional divergence. Different colored boxes with numbers show protein motifs.

To assess whether the sequence divergence among duplicates changed the proteins structures of syntenic *MAM* genes, we analyzed nine conserved motif patterns in 41 *MAM* proteins in the 12 Brassicaceae species using the MEME tool (Bailey et al., 2009) (Figure 5). Motifs 1, 2, 3, and 4 were identified to belong to the DRE_TIM_metallolyase super family [cl18962] conserved domain, and motifs 5, 6, 7, 8, and 9 were to belong to the PLN03228 (methylthioalkylmalate synthase) conserved domain reported by the Conserved Domain Search Service (Marchler-Bauer et al., 2011).

The *MAM* genes in lineage I species encode proteins with more conservative structures than those of proteins encoded by genes in *A*. *arabicum* and lineage II species. One *MAM* genes of *A. arabicum* encode protein with intact motifs, while the others lack one or two motifs. In lineage I, apart from *LaMAM*, *CsMAM-1*, and *CsMAM-7*, the other *MAM* genes all encode proteins with nine intact motifs. In lineage II, especially in the *Brassica* genus, three-fifths of the syntenic *MAM* genes of *B. rapa* and half of the *MAM* genes of *B. oleracea* and *R. sativus* encode proteins with incomplete motifs, suggesting that local TD and Br-α events led to major divergences in protein structure. The lost motifs were either [cl18962] or PLN03228 motifs for different *MAM* gene copies. Because enzymes with these motifs catalyze the first committed step in leucine biosynthesis or methionine biosynthesis, the lost motifs may result in lower enzyme activity, or inactive enzymes.

### Purifying selection events were detected and even more frequent than positive selection

Next, we inferred whether selection acted on the nine conserved motifs during the lineage-specific evolution of *MAMs*. We calculated the non-synonymous/synonymous substitution ratio (Ka/Ks) for each motif of above syntenic *MAM* genes using the tool KaKs Calculator (Zhang et al., 2006). Motifs 1, 2, and 5 showed an overall excess, while motifs 3, 4, 6, and 8 showed more than 95% excess of synonymous changes relative to non-synonymous changes (Ka/Ks<1) (Supplementary Table 3), indicating strong purifying selection. However, Ka/Ks analysis for motifs 7 and 9 of the overall syntenic *MAM* genes showed that more than 10% of the sequences had undergone rapid non-synonymous to synonymous substitutions (Ka/Ks>1), indicating positive selection. The excess of non-synonymous changes in these two motifs may reflect general variations in the 3D structural framework that differentiate the activities or function of *MAM* enzymes.

To test this hypothesis, we calculated the Ka/Ks ratio of motif 7 and motif 9 respectively of syntenic *MAM* genes of lineages I and lineage II species. We found that more than 80% ancestral-like genes (Figure 3, blue blocks) showed excess synonymous changes (Ka/Ks<1) (Table 2), indicating the general conservation of these two motifs. That is, the activity of the enzymes encoded by these genes was conserved. However, in motif 7 (Table 2), more than 80% of the lineage I ancestral-like genes vs. *MAMb*, 70% of the lineage II genes vs. *MAMb*, and 50% of the lineage II ancestral-like genes vs. *AtMAM3* showed a Ka/Ks of greater than 1, indicating an excess of non-synonymous changes. This result showed that positive selection had driven motif 7 of *MAMb* and *AtMAM3* (Figure 3, pink blocks) distinguished from the ancestral-like genes (Figure 3, blue blocks).

**Table 2:** **Statistical analysis of Ka/Ks for motif 7 and motif 9 of syntenic *MAM* genes responsible for short- and long-chain glucosinolate biosynthesis**.

	**Ka/Ks<1**		**Ka/Ks>1**	
Motif 7	*CrMAM-1*^a^	81.82%	*MAMb*^b^	83.33%
	*CsMAM-7*^a^	93.55%	*MAMb*^c^	70.00%
	*MAMa*^a^	100.00%	*AtMAM3*^c^	50%
Motif 9	*CrMAM-1*^a^	92.86%	*TsMAM-1*^c^	77.78%
	*CsMAM-7*^a^	100.00%	*ThMAM-2*^c^	77.78%
	*MAMa*^a^	100.00%	*SpMAM-1*^c^	44.44%

a *Indicated CrMAM-1 vs. other ancestral MAM genes (blue blocks in lineage I and lineage II species, Figure 3)*.

b *Indicated MAMb vs. lineage I ancestral MAM genes (blue blocks, Figure 3)*.

c *Represented MAMb vs. lineage II ancestral-like MAM genes (blue blocks, Figure 3). Superscript letters represented the same comparisons for motif 9*.

For species from lineage II, the split out of *Thellungiella* is prior to the origin of *Brassica* genus (Haudry et al., 2013). We calculated the Ka/Ks ratio for three relatively ancient *MAM* genes of *Thellungiella* vs. the other lineage II *MAM* genes to test the selection pressure. We found for the motif 9 (Table 2), more than 75%, 75%, and 44% of the ancestral-like genes in lineage II vs. *TsMAM-1*, *ThMAM-2*, and *SpMAM-1*, respectively, had a Ka/Ks greater than 1. This indicated that there was strong positive selection driving the divergence between the ancestral-like genes and their tandem duplicates in lineage II species (Figure 3, green blocks).

## Discussion

The MAMs encoded by the *MAM* gene cluster are central to the diversification of Met-derived aliphatic glucosinolates in crucifer species. We took advantage of the completely or partially sequenced genomes of 13 Brassicaceae species (*A. thaliana*, *A. lyrata*, *C. rubella*, *L. alabamica*, *C. sativa*, *B. rapa*, *T. salsuginea*, *S. parvula*, *T. halophila*, *S. irio*, *B. oleracea*, *R. sativus*, and *A. arabicum* (Initiative, 2000; Dassanayake et al., 2011; Hu et al., 2011; Wang et al., 2011b; Wu et al., 2012; Haudry et al., 2013; Slotte et al., 2013; Yang et al., 2013b; Kagale et al., 2014; Kitashiba et al., 2014; Liu et al., 2014) to investigate the evolution and diversification of *MAM* genes at specific *MAM* loci in Brassicaceae. We proposed that the syntenic loci of *MAM* gene, which underwent frequent tandem duplications, evolved via two independent lineage-specific routes after their divergence from *A. arabicum*. Our analyses indicate that positive selection has driven the diversification of *MAM* genes involved in ailphatic glucosinolates. These findings will help further study of the function of *MAM* genes in Brassicaceae species.

### A lineage I-specific chromosome rearrangement occurred near *MAM* loci

Genome polyploidization is an evolutionary process that plays a key role in generating the diversity of plant species and providing abundant genetic materials for the evolution or expansion of gene families (Hittinger and Carroll, 2007; Spillane et al., 2007). The chromosomal constitution of each organism is reflected by its karyotype. Each species has a particular number of chromosomes with unique sizes and shapes (Schubert and Lysak, 2011). The genomes of species in Brassicaceae comprise 24 genomic blocks (A–X, also known as ancestral karyotypes, AK) (Parkin et al., 2005; Schranz et al., 2006), which can be observed in the recently sequenced genomes of *A. lyrata*, *S. parvula*, and *B. rapa* (Dassanayake et al., 2011; Hu et al., 2011; Wang et al., 2011b), as well as in the genome of the model plant *A. thaliana* (Initiative, 2000), etc.

In all of the sequenced genomes of Brassicaceae, the *MAM* loci are located at the end of Q Block, linked to X Block. Strikingly, the Q and X Blocks are distributed on different chromosomes in *C. sativa*, *C. rubella*, and *A. lyrata*, but on the same chromosome in *A. thaliana* (Chr5) and in lineage II species. This arrangement is proof of a large-scale chromosomal rearrangement event that occurred in lineage I species before the divergence of *A. lyrata* and *A. thaliana* approximately 10 million years ago (Hu et al., 2011), but after their split from *A. arabicum*. The lineage-I-specific genome rearrangement can be used to reconstruct ancient karyotypes and to detect very old polyploidization events in other lineage I species (Semon and Wolfe, 2007). Even though a large chromosomal-scale rearrangement event occurred during speciation, the *MAM* loci were not deleted, but were retained with highly conserved syntenic arrangements (Figure 3). Their retention and high degree of conservation are consistent with the importance of the enzymes encoded by genes at the *MAM* loci in secondary metabolite biosynthesis.

### Diversified functions of *MAM* loci may result in different Met-derived aliphatic glucosinolates

Glucosinolates are secondary metabolites that are well known for their role in resistance to insects and pathogens, as well as for their cancer-prevention properties. There are large differences among glucosinolate profiles because of differences in their amino acid precursors. Our analyses of the *MAM* loci in the 13 sequenced species indicate that there were two independent lineage-specific patterns of evolution.

In the early diverged sister *A. arabicum*, the four identified *AaMAM* genes could explain the abundance of 3-methylsulfonylpropyl, 4-methylsulfonylbutyl, and 8-methylsulfinyloctyl aliphatic glucosinolates, produced via one, two, and six rounds of carbon-chain extension, respectively (Al-Shammary, 1987). Additionally, the distinct glucosinolate profiles suggest these *AaMAM* genes could have gained diverse functions to biosynthesize glucosinolates with different chain-length. However, these four *AaMAM* genes could not be distinguished from *MAM1* or *MAM3* in our phylogenetic analyses. However, one gene (*AaMAM-3*) that differed from the others was identified as the ancestor of those in the other Brassicaceae species. In future research, it will be useful to experimentally test their activities *in vivo* or *in vitro* to determine which ones are responsible for short chain elongation or long chain elongation, respectively.

In the model plant *A. thaliana* and its congener *A. lyrata*, the natural variations in *MAMs* were shown to determine the glucosinolate phenotypes (Kroymann et al., 2001, 2003; Textor et al., 2004; Benderoth et al., 2006; Heidel et al., 2006). In *C. sativa*, which retains a highly undifferentiated hexaploid genome structure, 12 *MAM* genes were identified; 3 were derived from *MAMa*, 3 from *MAMc*, and 6 from *MAMb*. The large-scale expansion of *CsMAM* genes, especially those originating from *MAMb*, could contribute to the large quantities of long-chain aliphatic glucosinolates such as glucoarabin (7C), glucocamelinin (10C), and 11-(methylsulfinyl)-undecylglucosinolate (11C) in *C. sativa* seeds (Berhow et al., 2013). In *C. rubella*, the four annotated syntenic *MAM* genes are responsible for the biosynthesis of different chain-length glucosinolates; therefore, glucosinolates with various chain-lengths should be detectable in the organs of this species.

The six sequenced species in lineage II contain abundant short-chain glucosinolates and trace amounts long-chain glucosinolates. This is consistent with the presence of syntenic *MAM* genes encoding enzymes responsible for short-chain glucosinolate biosynthesis. For example, in *Thellungiella*, two syntenic tandem *MAM* genes with a *MAMa* function (Figure 2) were annotated in *T. salsuginea*, *S. parvula*, and *T. halophila* (Table 1). The presence of these tandem *MAM* genes can account for the three abundant short-chain glucosinolates, allylglucosinolate (3C), 3-ethylsulphinylpropylglucosinolate (3C), and 3-methylthiopropylpropylglucosinolate (3C) identified in *Thellungiella* flowers, siliques, and seeds (Pang et al., 2009, 2012). The other long-chain glucosinolate (10MSD) present at trace levels in *T. salsuginea* and *S. parvula* should be biosynthesized by enzymes encoded by the non-syntenic genes *TsMAM-3* and *SpMAM-3*. However, in *T. halophila*, the two syntenic *MAM* genes are thought to encode enzymes involved in short-chain glucosinolate biosynthesis. Therefore, we proposed that the *T. halophila* genome should contain another non-syntenic *MAM* gene encoding an enzyme for long-chain aliphatic glucosinolate biosynthesis. In *Brassica*, which experienced an additional WGT event compared with the model plant *A. thaliana*, five syntenic *MAM* genes sharing *MAMa* functions encode biosynthetic enzymes for the most abundant short-chain glucosinolates. These genes are involved in the biosynthesis of gluconapin (4C), glucobrassicanapin (5C), and progoitrin (4C) in *B. rapa* (Padilla et al., 2007; Lou et al., 2008; Kim et al., 2010) and in the biosynthesis of the major aliphatic glucosinolates sinigrin (3C) and glucoiberin (3C) in *B. oleracea* (Cartea et al., 2008). In *R. sativus*, two syntenic *MAM* genes were identified that could be responsible for the biosynthesis of the three main short-chain glucosinolates: glucosisaustricin (2C), glucosisymbrin (3C), and glucoraphenin (4C) (Ediage et al., 2011).

Some lineage I species (e.g., *Lepidium sativum*, *Cardamine hirsuta*, and *Rorippa islandica*) and lineage II species (e.g., *S. irio*) lack Met-derived glucosinolates but are rich in Val-, Ile-, and Leu-derived glucosinolates or aromatic glucosinolates (Franzke et al., 2011). The results of our synteny analyses suggest that the *MAM* genes of *S. irio* were lost after its divergence from *Thellungiella*, resulting in the lack of aliphatic glucosinolates. Therefore, we speculate that *MAM* genes should also have been lost from *L. sativum*, *C. hirsute*, and *R. islandica* when the genetic backgrounds were altered or as a result of environmental adaptation.

### Positive selection drove the diversification of *MAM* loci in core Brassicaceae

Based on the results obtained here, we propose a scenario for the evolutionary history of the *MAM* loci (Figure 4) in the family Brassicaceae. In this scenario, all *MAM* genes in the core Brassicaceae group evolved from a shared ancestor with *A. arabicum (AaMAM-3)* but were subjected to lineage-specific evolutionary processes by positive selection.

In lineage I, before the speciation of *A. lyrata* and *C. rubella*, two *MAM* genes with different functions were generated in the ancestor by amplification and functional differentiation. Subsequently, the duplicated gene (e.g., that in *A. lyrata*, *C. rubella*, and *C. sativa*) or the ancient gene (e.g., that in *A. thaliana*) underwent further rounds of TD and functional divergence, giving rise to three *MAM* genes with distinct functions. During the lineage I evolutionary process, the most conservative motifs (motifs 1, 2, and 5) underwent strong purifying selection, allowing the proteins encoded by the ancestral *MAM* genes (Figure 3, blue blocks) to share the same or similar enzyme activity. However, between the ancestral genes and the duplicates, motif 9 exhibited an excess of non-synonymous relative to synonymous changes. This indicated that strong positive selection had forced the diversification of the function of enzymes encoded by *MAM* genes. Additionally, the Ka/Ks ratios of motif 7 were greater than 1 in the following pairs: *AtMAM3* vs. *CsMAM-3*, *AtMAM2* vs. *CsMAM-3*, *CsMAM-7* vs. *CsMAM-3* as well as *AtMAM1* vs. *CsMAM-9*, *AtMAM2* vs. *CsMAM-9*, *CsMAM-1* vs. *CsMAM-9*, and *CsMAM-7* vs. *CsMAM-9*. This result indicated that positive selection had driven the above genes (Figure 3, orange blocks) to diverge from the other *MAM* genes (Figure 3, blue and pink blocks). We found that three motifs (motifs 3, 7, and 9) of *AtMAM1* vs. *AtMAM2* had excess non-synonymous changes (data not shown), indicating strong positive selection of the gene *AtMAM1*, consistent with the results of a previous report (Benderoth et al., 2006).

For species *Thellungiella* and *Brassica* from lineage II, TD and minor sequence variations could have occurred frequently to generate two types of *MAM* genes, which encoded products with the same or similar functions, under strong positive selection. Besides motifs 7 and 9, which provide positive selection pressure for the lineage II homologs, in motif 8, the pairwise analysis of the Ka/Ks for *CsMAM-1* vs. *ThMAM-2* (Ka/Ks = 1.92) and for *CsMAM-1* vs. *TsMAM-1*(Ka/Ks = 1.92) showed that there were strong positive selection pressures forcing the formation of the *MAM* loci in lineage II species. In this study, we found that various gene duplications (e.g., WGD and TD), functional divergence, and positive selection of *MAM* loci, via two different lineage-specific evolutionary routes, have contributed to the diversification of glucosinolates.

Previous studies have reported that different glucosinolates are involved in resistance responses to different herbivores and pathogens (Kroymann et al., 2003; Clay et al., 2009) and in increasing plant fitness (Manzaneda et al., 2010). Some specific glucosinolates (e.g., glucoraphanin, glucoraphenin, 4C) show powerful cancer-preventive properties (Hecht, 2000). *MAM* genes play an essential role in the diversity of aliphatic glucosinolates. In this context, understanding the evolution of the *MAM* genes will help us to understand the specific functions of *MAM* genes in the recently sequenced Brassicaceae species. For example, based on our analyses of the phylogenetic and syntenic relationships, we can predict which genes are related to long-chain glucosinolate biosynthesis and which are related to short-chain glucosinolate biosynthesis in *C. sativa*, and then predict its glucosinolates profile. In *B. napus*, the main glucosinolate in the leaves and seeds was identified as alkenyl glucosinolate (3C); this information was used to enhance the quality of rapeseed (Mithen, 1992; Parkin et al., 1994). Consistent with that study, our results predicted that *BnMAM* genes encode enzymes catalyzing short-chain glucosinolate biosynthesis. Indeed, in the recently published *B. napus* genome, seven syntenic *BnMAM* genes were annotated on the recent allopolyploid genome (Chalhoub et al., 2014). Our results suggest that these seven genes encode enzymes involved in short-chain glucosinolate biosynthesis.

Thus, understanding the evolution of *MAM* genes is not only helpful for answering questions about the patterns of conservation and divergence of *MAM* genes and the forces driving their evolution, but also for predicting the function of *MAM* genes and the glucosinolate profiles in Brassicaceae species. Therefore, this information will be useful for altering the glucosinolate profiles of Brassicaceae crops.

## Author contributions

Xiaowu Wang, Feng Cheng, and Wencai Yang designed the research. Jifang Zhang performed the research and analyzed the data. Xiaobo Wang, Feng Cheng, Jian Wu, and Jianli Liang contributed new computational tools and data. Jifang Zhang and Xiaowu Wang wrote the article.

### Conflict of interest statement

The Associate Editor Tiegang Lu and Review Editor Xiao Han declare that, despite being affiliated to the same institution as authors Jifang Zhang, Xiaobo Wang, Feng Cheng, Jian Wu, Jianli Liang and Xiaowu Wang, the review process was handled objectively and no conflict of interest exists. The authors declare that the research was conducted in the absence of any commercial or financial relationships that could be construed as a potential conflict of interest.
