# Supplementary material for: Lineage-specific evolution of Methylthioalkylmalate synthases (MAMs) involved in glucosinolates biosynthesis
Source: Front Plant Sci. 2015 Feb 3;6:18. doi: 10.3389/fpls.2015.00018 (PMC4315028; doi:10.3389/fpls.2015.00018)
Supplement: Supplementary file 1 [file DataSheet1.PDF]

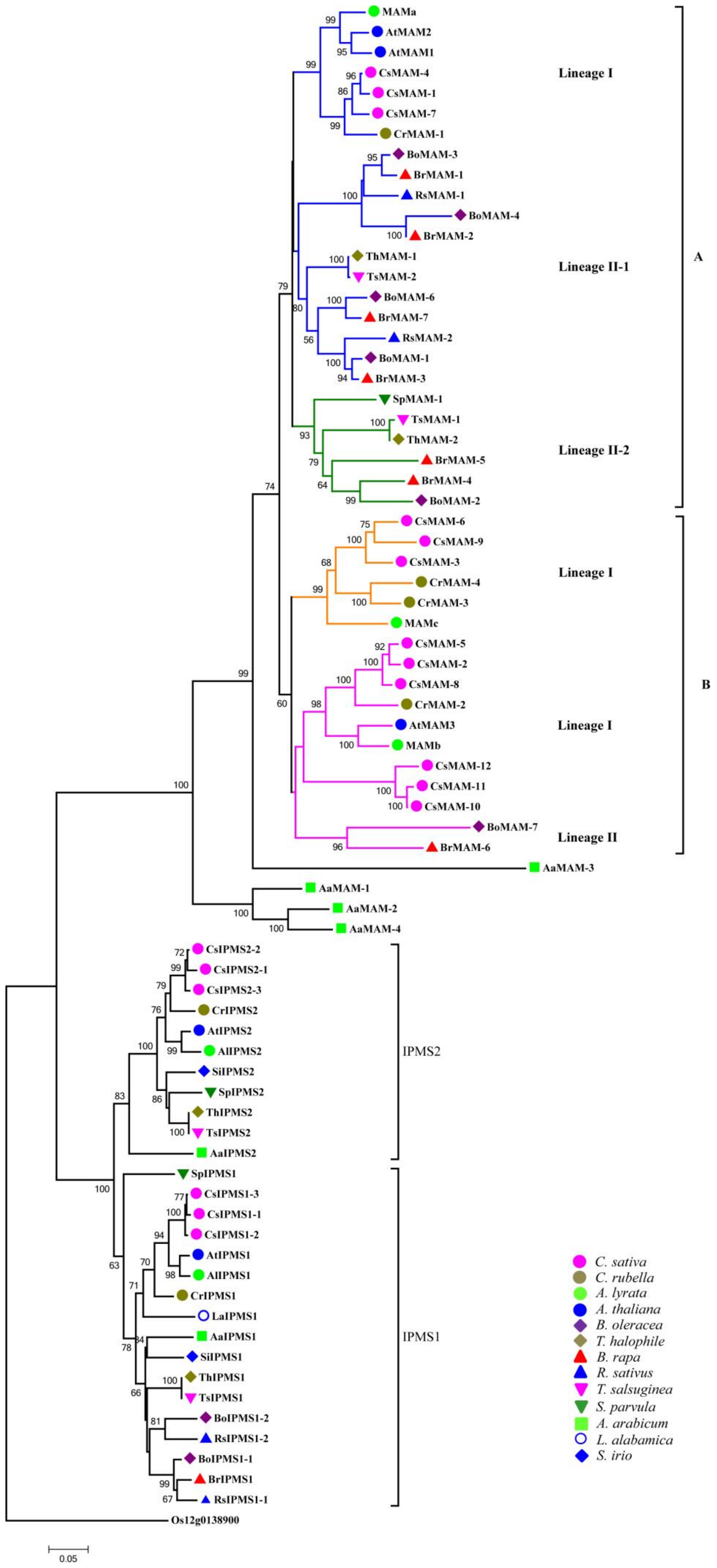

MAMa
AtMAM2
AtMAM1
CsMAM-4
CsMAM-1
CsMAM-7
CrMAM-1
BoMAM-3
BrMAM-1
RsMAM-1
BoMAM-4
BrMAM-2
ThMAM-1
TsMAM-2
BoMAM-6
BrMAM-7
RsMAM-2
BoMAM-1
BrMAM-3
SpMAM-1
TsMAM-1
ThMAM-2
BrMAM-5
BrMAM-4
BoMAM-2
CsMAM-6
CsMAM-9
CsMAM-3
CrMAM-4
CrMAM-3
MAMc
CsMAM-5
CsMAM-2
CsMAM-8
CrMAM-2
AtMAM3
MAMb
CsMAM-12
CsMAM-11
CsMAM-10
BoMAM-7
BrMAM-6
AaMAM-3
AaMAM-1
AaMAM-2
AaMAM-4
CsIPMS2-2
CsIPMS2-1
CsIPMS2-3
CrIPMS2
AtIPMS2
AlIPMS2
SiIPMS2
SpIPMS2
ThIPMS2
TsIPMS2
AaIPMS2
SpIPMS1
CsIPMS1-3
CsIPMS1-1
CsIPMS1-2
AtIPMS1
AlIPMS1
CrIPMS1
LaIPMS1
AaIPMS1
SiIPMS1
ThIPMS1
TsIPMS1
BoIPMS1-2
RsIPMS1-2
BoIPMS1-1
BrIPMS1
RsIPMS1-1
Os12g0138900
Lineage I
Lineage II-1
Lineage II-2
A
Lineage I
Lineage I
Lineage II
B
IPMS2
IPMS1
C. sativa
C. rubella
A. lyrata
A. thaliana
B. oleracea
T. halophile
B. rapa
R. sativus
T. salsuginea
S. parvula
A. arabicum
L. alabamica
S. irio
0.05

Supplemental Figure 1 Phylogeny relationships of *MAM* genes in Brassicaceae. Phylogenetic tree was constructed based on the full-length sequences of MAM and IPMS proteins identified in sequenced genomes of 13 Brassicaceae species. Four genes were excluded (*LaMAM*, *SpMAM-2*, *BoMAM-5* and *TsMAM-3*) because they had abnormal gene structures. Rice *IPMS* served as the outgroup. *IPMS*, encoding isopropylmalate synthase, which is involved in leucine biosynthesis, is thought to be the ancestor of *MAM* genes (de Kraker, 2007). *IPMS1* and *IPMS2* genes in Brassicaceae evolved independently after divergence from an ancestor.

Supplemental Table 1 *IPMS* genes used in the *MAM* phylogeny analysis

| **Species** | **Gene ID** | **Reference** |
|---|---|---|
| *A. arabicum* | *AaIPMS1* (AA_scaffold3815_40),<br>*AaIPMS2* (AA_scaffold2581_40) | Haudry et al. (2013a) |
| *C. rubella* | *CrIPMS1* (Carubv10008887m),<br>*CrIPMS2* (Carubv10019832m) | Slotte et al. (2013) |
| *L. alabamica* | *LaIPMS1* (LA_scaffold951_48) | Haudry et al. (2013a) |
| *C. sativa* | *CsIPMS2-1* (Csa07g044290.1),<br>*CsIPMS2-2* (Csa09g078640.1),<br>*CsIPMS2-3* (Csa16g037970.1),<br>*CsIPMS1-2* (Csa14g023830.1),<br>*CsIPMS1-3* (Csa17g024310.1),<br>*CsIPMS1-1* (Csa03g022350.1) | Kagale et al. (2014) |
| *T. halophila* | *ThIPMS1* (Thhalv10007073m),<br>*ThIPMS2* (Thhalv10018267m) | Yang et al. (2013) |
| *T. salsuginea* | *TsIPMS1* (Tsa1g16490),<br>*TsIPMS2* (Tsa5g31590) | Wu et al. (2012) |
| *S. parvula* | *SpIPMS1* (c0013_00070),<br>*SpIPMS2* (c0001_01433) | Dassanayake et al. (2011) |
| *S. irio* | *SiIPMS1* (SI_scaffold1491_333),<br>*SiIPMS2* (SI_scaffold242_257) | Haudry et al. (2013a) |
| *B. oleracea* | *BoIPMS1-1* (Bol009726),<br>*BoIPMS1-2* (Bol030767) | Liu et al. (2014) |
| *B. rapa* | *BrIPMS1* (Bra031031) | Wang et al. (2011) |
| *R. sativus* | *RsIPMS1-1* (Rsa10013839),<br>*RsIPMS1-2* (Rsa10038223) | Kitashiba et al. (2014) |

Supplemental Table 2 Conserved motifs identified by MEME using protein sequences of *MAM* genes from 12 Brassicaceae species

| Motif | Width | Best possible match | Conserved domain |
|---|---|---|---|
| **1** | 73 | HCHNDLGVATANTIAGICAGARQVEVTINGIGERSGNAPLEEVVMALKCRGEYVMDGVYTRIDT RQIMATSKM | [cl18962] |
| **2** | 80 | WPEYIPNKLPDKNYVRVFDTTLRDGEQAPGAALTPPQKLEIARQLAKLRVDIMEVGFPGSSEEEF ETVKTIAKTVGNEVD | [cl18962] |
| **3** | 62 | YVPVICAIARCKHRDIEAAWEAVKYAKRPRILIFTSTSDIHMKYKLKKTQEEVIEMAASSIR | [cl18962] |
| **4** | 57 | FAKSLGFNDIQFGCEDGCRSDKDFLCKILGEAIKAGATTVNFADTVGINMPHEYGEL | [cl18962] |
| **5** | 54 | DGMLKNRSTYEILSPEDIGIVKSQNSGIVLGKLSGRHAVKDRLKELGYEIDDEK | [PLN03228] |
| **6** | 29 | VQEYTGLYVQPHKPIVGANCFVHESGIHQ | [PLN03228] |
| **7** | 41 | SFPSLRLTRPYNKPSLFISCCSSVSKKAATSATDLKPIVER | [PLN03228] |
| **8** | 28 | LNDIFSRFRDLTKQKKRITDADLKALVT | [PLN03228] |
| **9** | 15 | YLKANTPGIDDVVFS | [PLN03228] |

[PLN03228], methylthioalkylmalate synthase.
[cl18962], DRE-TIM metallolyase superfamily.

Supplemental Table 3 Pairwise analysis of Ka/Ks for each motif of syntenic *MAM* genes

| | **Motif1 (%)** | **Motif2 (%)** | **Motif3 (%)** | **Motif4 (%)** | **Motif5 (%)** | **Motif6 (%)** | **Motif7 (%)** | **Motif8 (%)** | **Motif9 (%)** |
|---|---|---|---|---|---|---|---|---|---|
| **Ka/Ks>1** | 0.00 | 0.00 | 0.51 | 0.95 | 0.19 | 0.51 | 17.07 | 3.47 | 10.35 |
| **Ka/Ks=1** | 0.00 | 0.00 | 0.00 | 0.00 | 0.00 | 0.00 | 0.00 | 0.00 | 0.00 |
| **Ka/Ks<1** | 100 | 100 | 99.49 | 99.05 | 99.81 | 99.49 | 82.93 | 96.53 | 89.65 |
